# Novel Mutations Detection with Next-Generation Sequencing and Its Association with Clinical Outcome in Unilateral Primary Aldosteronism

**DOI:** 10.3390/biomedicines9091167

**Published:** 2021-09-06

**Authors:** Che-Hsiung Wu, Kang-Yung Peng, Daw-Yang Hwang, Yen-Hung Lin, Vin-Cent Wu, Jeff S. Chueh

**Affiliations:** 1 Division of Nephrology, Taipei Tzu Chi Hospital, Buddhist Tzu Chi Medical Foundation, New Taipei City 231, Taiwan; tcubear@gmail.com; 2 School of Medicine, Tzu Chi University, Hualien 970, Taiwan; 3 Taiwan Primary Aldosteronism Investigation (TAIPAI) Study Group, Taipei 100, Taiwan; 4 Division of Nephrology, Department of Internal Medicine, National Taiwan University Hospital, Taipei 100, Taiwan; pengky68@gmail.com; 5 Division of Nephrology, Kaohsiung Medical University Hospital, Kaohsiung Medical University, Kaohsiung 807, Taiwan; 910208@kmuh.org.tw; 6 Division of Cardiology, Department of Internal Medicine, National Taiwan University Hospital, Taipei 100, Taiwan; austinr34@gmail.com; 7 Department of Urology, and College of Medicine, National Taiwan University Hospital, Taipei 100, Taiwan; jeffchueh@gmail.com

**Keywords:** next-generation sequencing, unilateral primary aldosteronism, outcome

## Abstract

Somatic mutations have been identified in adrenal tissues of unilateral primary aldosteronism (uPA). The spectrum of somatic mutations in uPAs was investigated using a customized and targeted next-generation sequencing (cNGS) approach. We also assessed whether cNGS or Sanger sequencing-identified mutations have an association with clinical outcomes in uPA. Adrenal tumoral tissues of uPA patients who underwent adrenalectomy were obtained. Conventional somatic mutation hotspots in 240 extracted DNA samples were initially screened using Sanger sequencing. A total of 75 Sanger-negative samples were further investigated by sequencing the entire coding regions of the known aldosterone-driver genes by our cNGS gene panel. Somatic mutations in aldosterone-driver genes were detected in 21 (28%) of these samples (8.8% of all samples), with 9 samples, including mutations in *CACNA1D* gene (12%), 5 in *CACNA1H* (6.6%), 3 in *ATP2B3* (4%), 2 in *CLCN2* (2.6%), 1 in *ATP1A1* (1.3%), and 1 in *CTNNB1* (1.3%). Via combined cNGS and Sanger sequencing aldosterone-driver gene mutations were detected in altogether 186 of our 240 (77.5%) uPA samples. The complete clinical success rate of patients containing cNGS-identified mutations was higher than those without mutations (odds ratio (OR) = 10.9; *p* = 0.012). Identification of somatic mutations with cNGS or Sanger sequencing may facilitate the prediction of complete clinical success after adrenalectomy in uPA patients.

## 1. Introduction

Primary aldosteronism (PA) is characterized by excessive aldosterone production and affects 5–20% of hypertensive patients [1,2]. The mechanisms causing excessive aldosterone production have been explored via the identification of somatic and germline mutations in PA patients. Studies have identified somatic mutations in the majority of aldosterone-producing adenomas (APAs). Mutations in the selectivity filter of the potassium channel GIRK4 (encoded by *KCNJ5*) abolish potassium selectivity and increase sodium entry and membrane depolarization [3]. Somatic mutations in an L-type voltage-gated Ca^2+^ -channel subunit, Cav1.3 (encoded by *CACNA1D*), and two ATPases (Na+/K+ ATPase Alpha-1 Subunit and Ca^2+^ ATPase 3, encoded by *ATP1A1* and *ATP2B3*, respectively) were also identified [4,5,6]. In addition to ion channels and ATPase, some APAs carry gain-of-function mutations in *CTNNB1* encoding β-catenin with multiple important cellular functions, including activation of WNT signaling, identified in 2–5% of APA patients [7,8]. Germline mutations in *CLCN2*, encoding the voltage-gated chloride channel protein ClC-2, were previously recognized in familial hyperaldosteronism (FH)-II [9]. A somatic *CLCN2* mutation was recently identified in a sporadic APA as well [10]. A somatic mutation in *CACNA1H*, encoding a voltage-dependent T-type calcium channel alpha-1H subunit mutated in FH type IV, was also identified in an APA using whole-exome sequencing lately. The prevalence of somatic *CACNA1H* mutations in APA was relatively low in the study cohort (~4%) [11]. Mutations in these genes cause increased intracellular calcium concentrations, activation of calcium signaling, and thereby increased aldosterone biosynthesis.

Previous studies assessed only selected exons or hotspot regions of these genes, and the prevalence of APA somatic mutations was estimated at around ~50% [12], with a high proportion of tumors without known mutations [13]. However, major advances in next-generation sequencing (NGS) allowed further identification of novel somatic mutations that might be related to excessive aldosterone production in APA. A recent study by De Sousa et al. using CYP11B2 immunohistochemistry as a tool to guide the identification of mutations in conjunction with NGS demonstrated that as high as 93.75% of adenomas had an aldosterone-driving mutation [14]. A higher percentage of *CACNA1D* mutation (>40%) in the black population has also been identified by the targeted NGS approach [15]. Thus, the identification of somatic mutations enables the detection of PA subtypes with distinct pathological features and clinical presentations. Here, we investigated the prevalence of somatic mutations in sporadic unilateral PA tumoral tissues from the Taiwan Primary Aldosteronism Investigation (TAIPAI) tissue bank via a cNGS analysis targeting genes frequently mutated in APAs. In addition, previous studies demonstrated that KCNJ5 mutation carriers have a higher likelihood of cure from hypertension after adrenalectomy [16,17]. However, little is known about the association of other mutations in aldosterone-driver genes and the clinical outcomes after adrenalectomy in uPA patients. In this study, we also intended to evaluate whether cNGS or Sanger sequencing-identified mutations have an association with clinical outcomes in uPA.

## 2. Materials and Methods

### 2.1. Study Population

The inception cohort was based on the Taiwan Primary Aldosteronism Investigation (TAIPAI) database and tissue bank. The TAIPAI registry was constructed for quality assurance in 2 medical centers, 3 affiliated hospitals, and 2 regional hospitals in different cities of Taiwan [18,19]. Anti-hypertensive medications were discontinued for at least 21 days before confirmatory tests. When necessary, diltiazem and/or doxazosin were administered to control markedly high blood pressure [20]. Patients with an abnormal aldosterone-renin ratio (ARR) were confirmed as PA by saline infusion or captopril tests. Subtypes of PA were identified via imaging (Supplementary Figure S1). Criteria for uPA identification were [20]: (1) confirmed PA diagnosis; (2) imaging evidence for a unilateral adrenal adenoma or hyperplasia; (3) lateralization of aldosterone secretion with adrenal vein sampling (AVS) or during dexamethasone suppression NP-59 SPECT/CT [21] to the above-mentioned imagine-finding side.

The study included adrenal tumoral tissues from 240 prospectively recruited patients with uPA who underwent adrenalectomy between March 2011 and June 2017. The unilateral adrenalectomy of the lesion-side adrenal gland was performed via the lateral transperitoneal laparoscopic approach by experienced surgeons. Excised adrenal tumors were freshly frozen and stored at −80 °C. First, conventional somatic mutation hotspots of *KCNJ5, ATP1A1, ATP2B3, CLCN2*, and *CTNNB1* in DNA samples from the representative APA tissue sections with the largest circumscribed encapsulated tumor area and with a distinctive golden-yellow cut surface were sequenced using the Sanger method. Sanger Sequencing was performed by the Sequencing Core, Department of Medical Research, National Taiwan University Hospital. CACNA1D and CACNA1H are large genes and are difficult to sequence in entirety by Sanger sequencing. Therefore, we proceeded directly to cNGS for these 2 genes. All primer sequences are indicated in Supplementary Table S1. The remaining adenomas without known mutations via the Sanger method were further investigated by NGS.

### 2.2. Mutation Analysis

Genomic DNA was extracted from excised adenomas using a QIAamp DNA mini kit (Qiagen, Hilden, Germany). A customized aldosterone-driving gene panel was used, including 7 genes: *KCNJ5*, *ATP1A1*, *ATP2B3*, *CACNA1D*, *CACNA1H*, *CLCN2*, and *CTNNB1.* All coding exons and at least 10 base pair-long (bp) flanking sequences at intron-exon boundaries were amplified using targeted specific primers. A Multiplexed PCR-based library was prepared using a Fluidigm Access-Array [22]. Primer pools were generated per PCR with a final concentration of 1 μM per primer. Each sample master mix contained 50 ng genomic DNA, 5% dimethyl sulfoxide, 1x FastStart High Fidelity Reaction Buffer with MgCl_2_, FastStart High Fidelity Enzyme Blend, dNTPs (200 mM each), and 1x Access Array loading reagent (Roche, Indianapolis, IN, USA). The total of 48 different DNA samples was mixed with 48 different 4 to 5-plex primer (readjust due to gene list) pools on one 48.48 Access Array followed by thermal cycling. Harvested amplicon pools received another PCR step to barcode the products based on the manufacturer’s protocol. Barcoded PCR products were pooled, and re-sequenced using an Illumina MiniSeq NGS platform. Accession numbers of the genes: KCNJ5: NM_000890; ATP1A1: NM_001160233.2; ATP2B3: NM_001001344.3; CACNA1D: NM_000720.4; CACNA1H: NM_001005407.2; CLCN2: NM_004366.6; CTNNB1: NM_001904.

### 2.3. Bioinformatics Analysis

CLCbio Genomics Workbench 10 (Qiagen) was used. Filtering criteria were as follows: (1) exclude minor variant frequency <10%; (2) exclude dbSNP150 with minor allele frequency >1%; (3) keep non-synonymous changes and splice-site and frameshift variants; (4) keep variants with minor variant frequency >30%. Variants presented in the Exome Variant Server (EVS, http://evs.gs.washington.edu/EVS/; Accessed: 18 April 2020), Thousand Genome Project (TGP, http://www.internationalgenome.org/; Accessed: 18 April 2020), more than 5 hits in the Genome Aggregation Database (gnomAD, http://gnomad.broadinstitute.org; Accessed: 18 April 2020), and more than 1 hit in the Board Institute Exome Aggregation Consortium (ExAC, http://exac.broadinstitute.org; Accessed: 18 April 2020) were excluded [23]. For previously-unidentified somatic mutations in APA, Combined Annotation Dependent Depletion (CADD v1.2) [24], Polymorphism Phenotyping v2 (PolyPhen-2 v2.2.2 build r394) [25], Sorting Intolerant From Tolerant (SIFT v1.03) [26], and the Mutation Significance Cutoff (MSC) generated by the CADD (MSC-CADD) [24] and PolyPhen-2 scores (MSC-PolyPhen2) [25] were used to prioritize variants. CADD cutoff value was 20. Only variants that do not against more than one of the five in silico tool as probably damaging were considered as novel mutations. No NGS identified mutations were found in peripheral blood mononuclear cells of the patient.

### 2.4. Pathogenicity Criteria for Filtered Variants

The confirmed rare variants present in disease databases (HGMD, http://www.hgmd.org/; Accessed: 22 April 2020; ClinVar, https://www.ncbi.nlm.nih.gov/clinvar/; Accessed: 22 April 2020; LOVD, http://www.lovd.nl/; Accessed: 22 April 2020) and null variants (including frameshift, nonsense, obligatory splicing site, and initial codon mutations) identified in known APA genes, where gain of function is a known disease mechanism, were considered probably disease-causing [27].

### 2.5. Clinical Parameters

The arterial stiffness via brachial-ankle pulse wave velocity (baPWV) was evaluated with the subject in a supine position after a 15 min rest using an automatic waveform analyzer (Colin VP-2000, Omeron Inc., Kyoto, Japan) [28]. Occlusive cuffs connected to oscillatory and plethysmographic sensors were wrapped around ankles and the upper arms to measure blood pressure and analyze pulse waveforms. Cystatin C was measured using a particle-enhanced immunonephelometric assay (N Latex Cystatin C; Siemens, Berlin, Germany) with a nephelometer (BNII; Siemens, Berlin, Germany). Daily protein loss was defined as the urinary microalbumin-to-creatinine ratio (mg/mg). We separately measured the baPWV for both sides twice and calculated the mean baPWV for each side. The final baPWV was defined as the maximum of the values for right and left side baPWVs.

### 2.6. Clinical and Biochemical Success after Unilateral Adrenalectomy

Patients were evaluated monthly during the first 3 months after the operation and every 3 months thereafter. Complete clinical success (hypertension-remission) was defined as normalized blood pressure without anti-hypertensive use 12 months after adrenalectomy. Partial clinical success was defined as the same blood pressure as before surgery with less anti-hypertensive medication or a reduction in blood pressure with either the same amount or less anti-hypertensive medication [29]. Complete biochemical success was defined as normalized serum potassium levels (≥3.5 mmol/L) and ARR. Partial biochemical success was defined as the correction of hypokalemia and a raised aldosterone-to-renin ratio with one or both of the following (compared with pre-surgery): abnormal but improved post-surgery confirmatory test result; or ≥50% decrease in baseline plasma aldosterone concentration according to the Primary Aldosteronism Surgery Outcome (PASO) criteria on clinical and biochemical consensus [29] (Supplementary Table S2).

### 2.7. Tissue Immunohistochemistry

Immunohistochemistry (IHC) was conducted using mouse monoclonal antibody for CYP11B2 and rat monoclonal antibody for CYP11B1 (generous gifts from Professor Celso Gomez-Sanchez [30]). Sections of paraffin-embedded adrenal tumor and surrounding tissues were stained using the non-biotin-amplified method (Novolink; Novocastra Laboratories Ltd., Newcastle Upon Tyne, UK) according to the manufacturers’ protocol. Images were acquired using Olympus BX51 fluorescence microscope with a built-in Olympus DP72 camera and processed using cell Sens Standard 1.14 software (Olympus, Hamburg, Germany).

### 2.8. Statistical Analysis

Normally-distributed continuous variables were presented as mean ± standard deviation and analyzed using Student’s *t* test. Group differences were analyzed using Mann–Whitney U and Chi-square or Fisher’s exact tests for quantitative non-normally distributed and categorical variables, respectively. Multinomial logistic regression was performed to identify clinical outcome determinants. R version 4.0 and SPSS Statistics 19.0 were used in all analyses. *p* < 0.05 was considered statistically significant.

## 3. Results

### 3.1. Identification of Somatic Mutations in uPA

Conventional somatic mutations in *KCNJ5*, *CTNNB1*, *ATP1A1,* and *ATP2B3* were identified via the Sanger sequencing method of 240 DNA samples extracted from uPA tumoral tissues as the first step. We identified mutations in *KCNJ5*, *CTNNB1,* and *ATP1A1* in 151 (62.9%), 10 (4.1%), and 4 (1.7%) adrenal tumoral samples, respectively. The remaining 75 Sanger-negative samples were further investigated via our cNGS method, and 21 (28% of 75; 8.8% of all 240 samples) additional somatic mutations were identified (Table 1).

Reference sequences used for the variant calling: NM_001160233.2 for ATP1A1; NM_001001344.3 for ATP2B3; NM_000720.4 for CACNA1D; NM_001005407.2 for CACNA1H; NM_004366.6 for CLCN2. Abbreviations: Alt, alternative allele; Chr, chromosome; CADD, Combined Annotation Dependent Depletion; D, deleterious; M-C, MSC-CADD Score; MSC, Mutation Significance Cutoff; n/a, not available; P, probably damaging; Pred, prediction; PP2, PolyPhen2; Ref, reference Allele; SIFT, Sorting Intolerant From Tolerant; T, tolerated; VAF, Variant Allele Frequency (%).

**Figure 1:**
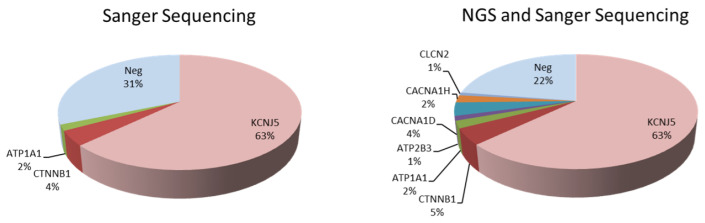
Frequency of genetic mutations identified in unilateral primary aldosteronism. Abbreviations: NGS, next-generation sequencing.

Hence, the total frequency of somatic mutations in the tumoral tissues among our uPA patients was 77.50% (186 mutations identified in 240 samples). Highest prevalence was observed in *KCNJ5* (*n* = 151, 63%), followed by *CTNNB1* (*n* = 11, 5%), *CACNA1D* (*n* = 9, 4%), *ATP1A1* (*n* = 5, 2%), *CACNA1H* (*n* = 5, 2%), *ATP2B3* (*n* = 3, 1%), and *CLCN2* (*n* = 2, 1%) (Figure 1).

**Figure 2:**
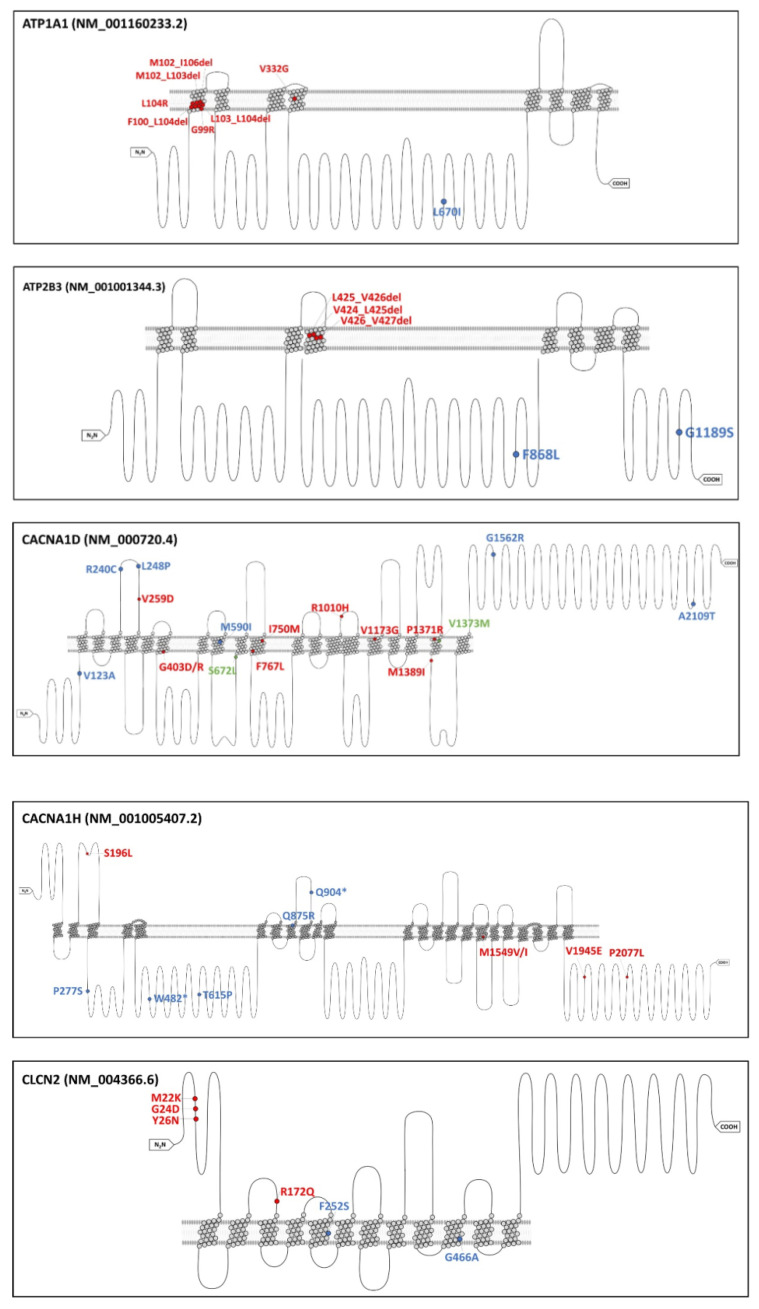
Genetic mutations identified in unilateral primary aldosteronism via targeted next-generation sequencing. Proteins were visualized with a Protter tool (Bioinformatics, Volume 30, Issue 6, 15 March 2014, Pages 884–886). Red font indicated the known hotspot mutation position that has been confirmed to affect electrophysiological properties or to affect aldosterone production in vitro. Blue font indicated genetic mutations identified in unilateral primary aldosteronism via targeted next-generation sequencing (as listed in Table 1). Green font indicated both of the above.

Out of our cNGS-identified somatic mutations, six *CACNA1D* mutations (p.Val123Ala, p.Arg240Cys, p.Leu248Pro, p.Met590Ile, p.Gly1562Arg, and p.Ala2109Thr), five *CACNA1H* mutations (p.Pro277Ser, p.Trp482Ter, p.Thr615Pro, p.Gln875Arg, and p.Gln904Ter), two *ATP2B3* mutations (p.Phe868Leu and p.Gly1189Ser), two *CLCN2* mutations (p.Phe252Ser and p.Gly466Ala), one *CTNNB1* mutation (Gln123Ter.), and finally one *ATP1A1* mutation (p.Leu670Ile) were not previously reported in aldosterone-driver gene (Figure 2). Gly1189Ser (*ATP2B3*) mutation was detected in two samples, whereas the rest were found in only one sample each.

### 3.2. Phenotypic Characteristics of uPA Patients with Negative Sanger Sequencing Results

**Figure 3:**
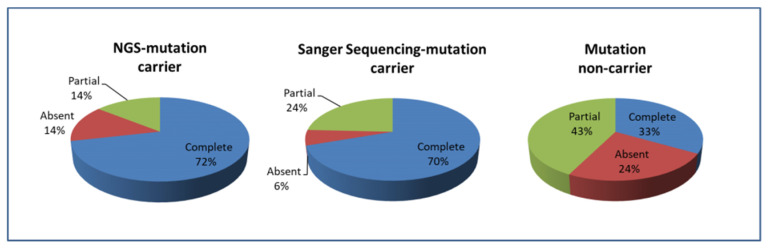
Clinical outcome for 75 patients with unilateral primary aldosteronism who underwent adrenalectomy with respect to detected mutations via customized, targeted next-generation sequencing. Definition of postoperative outcome: evaluation was performed at 12 months postoperatively. Clinical success was defined as resolution of hypertension and no need for anti-hypertensive medications.

The mean age of these 75 patients with negative Sanger sequencing results was 54.5 ± 10.6 years. The proportion of women was 57.3%. Carriers and non-carriers of NGS-identified mutated genes showed similar age, gender, baseline systolic and diastolic blood pressure, biochemistry data, tumoral size, and baPWV profiles (Table 2). Nonetheless, complete clinical success (i.e., hypertension –remission without medications at 1 year after adrenalectomy) rate of carriers of NGS-identified mutations (15 out of 21, 71.4%) was similar as that of carriers of Sanger sequencing-identified mutations (115 out of 165, 69.7%, *p* = 0.271). The complete clinical success rate of carriers of NGS and Sanger sequencing-identified mutations were significantly higher compared to that of those non-carriers (18 out of 54, 33.3%, *p* = 0.01 and <0.0001, respectively) (Figure 3). The genotype-phenotype correlation was further assessed by adding Sanger-identified mutation carrier in the analysis as the mutation carrier group to investigate the clinical significance of aldosterone-driver mutations (Supplementary Table S3). The complete clinical success rate of carriers of [NGS+Sanger]-identified mutations (130 out of 186, 69.9%) was significantly higher compared to that of those non-carriers (18 out of 54, 33.3%, *p* < 0.0001) (Supplementary Figure S2). Multinomial logistic regression analysis showed that NGS-identified mutation carrier status was the only independent predictor of complete clinical success (odds ratio (OR) = 10.9; 95% confidence interval (CI), 1.7–69.2; *p* = 0.012; Table 3). The final model was a significant improvement in fit over a null model (X^2^(22) = 38.99, *p* = 0.014) and showed a good fit with the data (Pearson and Deviance Chi-Square tests yielded *p* = 0.086 and *p* = 0.592, respectively). The overall predictive accuracy was 68%. Sensitivity analyses with models adding Sanger-identified mutation carrier in the analysis as the mutation carrier group and models adjusted for different clinical co-variables showed consistent results (Supplementary Tables S4 and S5). Furthermore, we made comparisons between the Sanger-positive group and the mutation-negative group to identify clinical outcome determinants using multinomial logistic regression. The presence of a mutation detected by Sanger sequencing was also predictive for the clinical outcome (OR = 4.1; 95% CI, 1.4–12.4; *p* = 0.015; Supplementary Table S6).

### 3.3. Histopathologic Characteristics of Adrenal Tumoral Tissues of uAPA

**Figure 4:**
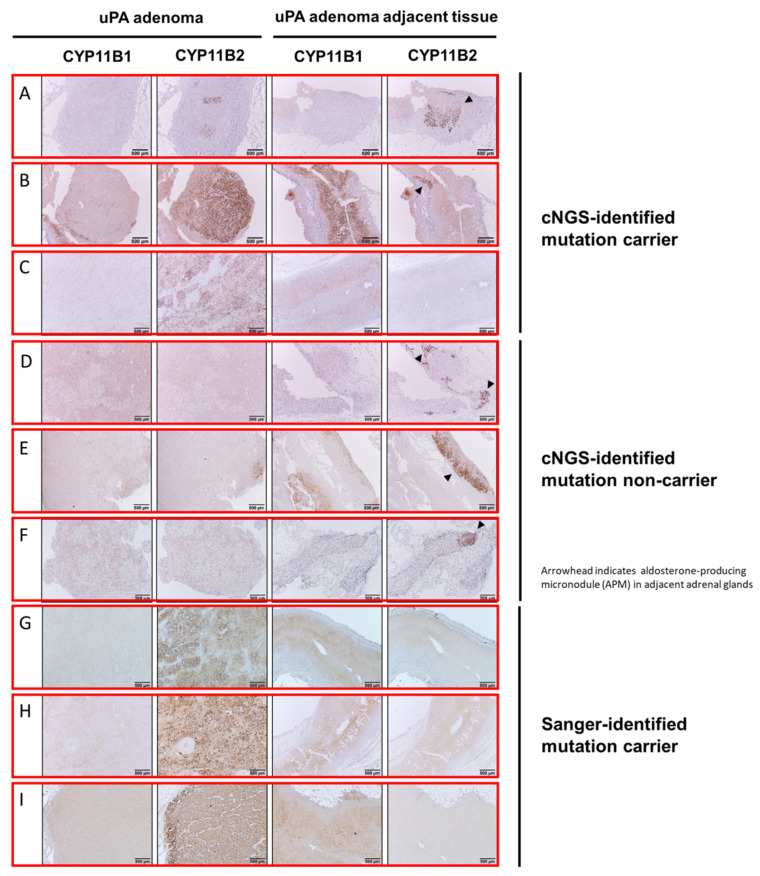
Histopathologic finding of tumoral and adjacent tissues of unilateral primary aldosteronism with respect to detected mutations via customized targeted next-generation sequencing. Higher magnification view of aldosterone-producing adenoma (**A**,**B**,**C**,**E**), and the CYP11B2-negative adenoma (**D**,**F**) with adjacent tissue. CYP11B2 and CYP11B1 IHC were quite variable among cNGS-identified mutation carriers and non-carriers. Arrowhead indicates aldosterone-producing micronodule (formally known as aldosterone-producing cell cluster) in adjacent adrenal glands. At least one aldosterone-producing micronodule could be found in tissues adjacent to the adrenal tumor. Scale bar: 500 μm. Abbreviations: cNGS, customized targeted next-generation sequencing; uPA, unilateral primary aldosteronism. For these Sanger sequencing-identified mutation carriers, CYP11B2 staining was homogeneous within adenoma (**G**–**I**).

The results of immunohistochemistry stain were quite variable among our cNGS-identified mutation carriers and non-carriers in regard to CYP11B2 expression. CYP11B2-stained adrenocortical tumor was detected in 51 (68%) of 75 adrenals with uPA; 11 with cNGS-identified mutations and 40 without. Of the remaining 24 (32%) adrenal glands with CYP11B2-negative adrenocortical tumors, 10 were associated with cNGS-identified mutation carriers and 14 had no cNGS-identified mutation (Supplementary Figure S3). All these 24 non-APA had CYP11B2-stained cell clusters at the subcapsular portion of peri-tumoral regions or adjacent para-tumoral tissue, in term of aldosterone-producing nodules (APN) or APM according to the HISTALDO (histopathology of primary aldosteronism) consensus [31]. For those 11 cNGS-identified mutation carriers, CYP11B2 staining could be homogeneous or heterogeneous within adenoma. The CYP11B1 IHC staining of cNGS-identified mutation carriers demonstrated that the density of CYP11B1 staining was similar between tumor and adjacent tumoral regions. The representative IHC images were illustrated in Figure 4A–F. The CYP11B2-stained adrenocortical tumor was detected in 100% of 165 adrenals with uPA harboring Sanger sequencing-identified mutations. For these Sanger sequencing-identified mutation carriers, CYP11B2 staining was homogeneous within adenoma (Figure 4G–I).

Subgroup analysis analyzing 51 uPA with CYP11B2-stained adrenocortical tumors showed outcomes consistent with our main results. Again, multinomial logistic regression analysis revealed that cNGS-identified mutation carrier status was the only independent predictor of complete clinical success (OR = 481.9; 95% CI, 2.4–98216.0; *p* = 0.002; Supplementary Table S7). The final model was a significant improvement in fit over a null model (X^2^(22) = 41.00, *p* = 0.008), and showed a good fit with the data (Pearson and Deviance Chi-Square tests yielded, *p* = 0.031 and *p* = 0.813, respectively). The overall predictive accuracy was 73.3%.

## 4. Discussion

Somatic mutations in uPAs were previously detected in approximately half of uPA tumoral tissues using conventional hotspot Sanger sequencing methodology [12]. Here, we used a customized and targeted NGS panel to investigate the prevalence of somatic mutations in sporadic uPA patients and achieved a nearly 80% (77.5%) detection rate for aldosterone-driver gene mutations. Several novel mutations in aldosterone-driver genes were identified by cNGS in our cohort. It is worth mentioning that mutations in CACNA1D were the most frequent NCS-identified somatic mutations in our cohort, which was similar to the finding of a Japanese cohort [32]. The prevalence of ATP1A1 mutations was lower (2%), yet similar to that for CACNA1H. CLCN2 and ATP2B3 mutations were the least-common mutations (~1%) in our cohort.

We demonstrated that the complete clinical success rate of patients containing our cNGS or Sanger sequencing-identified mutations was higher than those without any mutations. We also revealed that in uPA patients with CYP11B2 positive stained adenomas (true APA), the occurrence of the NGS-identified aldosterone-driver gene mutations were highly indicative of complete clinical success after adrenalectomy. Our results suggest that the presence of somatic mutations in uPA may be an indicator of better clinical outcomes after adrenalectomy. *KCNJ5* mutation carriers were shown to have a higher likelihood of cure from hypertension after adrenalectomy [16,17]. However, little is known about the association of other mutations in aldosterone-driver genes and the clinical outcomes after adrenalectomy in uPA patients. We herein showed for the first time that carriers with somatic mutations other than *KCNJ5* are also more likely to show complete clinical success after adrenalectomy based on PASO criteria.

A key aspect of our study is the cNGS analysis of a targeted panel of genes commonly mutated in uPA, which identified somatic mutations in aldosterone-driver genes in about 30% of samples, among which no mutations were detected by conventional Sanger sequencing. This is in accordance with recent publications on somatic mutations in aldosterone-regulating genes in uPA [14,15]. In contrast to smaller genes such as *KCNJ5*, large genes such as *CACNA1H* are difficult to sequence in their entirety, and only a limited number of mutations were previously found. Our cNGS successfully identified novel somatic mutations in these large genes. On the other hand, the two *KCNJ5* somatic mutations (p.Gly151Arg and p.Leu168Arg) account for 98% of known *KCNJ5* somatic mutations in APAs [12]; our cNGS did not identify further mutations.

Somatic mutations in *KCNJ5* were detected in approximately 60% of our all uPA patients, whereas an additional 5% and 4% included somatic mutations in *CTNNB1* and *CACNA1D*, respectively. The prevalence of *KCNJ5* mutation in this study lies between those of Western societies (~40%) [12] and Japanese (79%) [33] populations. Potential reasons of this difference include ethnic background, possible different selection criteria, or environmental factors.

No mutations were identified by NGS in some CYP11B2 positive tumors. This may be due to the strict criteria we used to define previously unidentified mutations. Only variants that do not against more than one of the five in silico tools (CADD, PolyPhen-2, SIFT, MSC-CADD, MSC-PolyPhen2) as probably damaging were considered as novel mutations. Among those 24 CYP11B2-negative adrenal tumors, at least one APN or APM could be found in tissues adjacent to the adrenal tumor. This re-demonstrates that all recruited cases in our cohort fulfilled the diagnostic criteria of uPA according to HISTALDO consensus [15]. Intriguingly, mutations in aldosterone-driving genes were still observed in 10 of those 24 CYP11B2-negative adrenal tumors in our cohort. It has been shown that *KCNJ5* could be sufficient to cause APA formation since *KCNJ5* mutations not only induce increased aldosterone production but it is also associated with adrenal cells proliferation [34]. CYP11B2 expression was supposed to be positive in true adenomas and be negative in non-functional adenomas. However, De Sousa et al. [14] found that sequenced areas showing *KCNJ5* mutations could exhibit heterogeneous expressions of CYP11B2, i.e., *KCNJ5* mutation could be identified in CYP11B2 positive or negative adrenal tumors, which may suggest repression of CYP11B2 activity in certain functional tumors by undisclosed mechanisms. In the same study, a *CACNA1D* mutation was identified in CYP11B2 negative region of possible aldosterone-producing cell cluster-to-APA transitional lesions (pAATLs). Moreover, Nanba et al. demonstrated that somatic *CTNNB1* mutation was observed both in CYP11B2-positive and -negative regions^34^. These data suggested that among certain uPA adrenal tissues, CYP11B2 may not be expressed even if there exist mutation(s) in aldosterone-driver genes. One possibility is that there might be dynamic functional statuses of aldosterone-producing cells with alternations between low and high CYP11B2 expression. Another possibility is that mutation was detected in contaminated DNA from APM adjacent to CYP11B2-negative tumors since DNA was isolated from macroscopically excised adrenal tissue. APM is frequently observed in adjacent adrenal to APA, and highly sensitive NGS may pick up variants in contaminated APM DNA. However, our subgroup analysis analyzing only uPA with CYP11B2-stained adrenocortical tumors showed outcomes consistent with the main results. Further large-scale investigations will be warranted to determine the impact of CYP11B2 heterogeneity on somatic mutation study among uPA.

Growing evidence supports a two-hit APA formation model. The first hit drives nodule formation, whereas somatic mutations in aldosterone biosynthesis genes provide the secondary hit [35]. APA tumorigenesis was proposed to occur within preexisting nodules through the acquisition of somatic mutations driving aldosterone production [36]. In patients with aldosterone-driver gene mutations, unilateral adrenalectomy of the prominent site could halt aldosterone overproduction and, therefore, either cure or significantly improve hypertension. On the other hand, aldosterone overproduction mechanisms in patients without mutation are still unknown. Surgical removal of these adrenal glands containing mutation-negative tumors led to a lower success rate (33.3% versus 71.4% of NGS-identified mutation-containing uPA patients) of hypertension remission in our series. Therefore, obtaining a comprehensive somatic mutation status in APA patients may help predict long-term clinical outcomes after surgery. This finding also brings up a concern about the application of adrenal-sparing surgery in patients with uPA. Identification of specific characteristics or surrogate biomarkers of somatic mutation status may allow further targeted treatment options preoperatively.

We identified several novel mutations in aldosterone-driver genes via our cNGS method. Sanger sequencing is limited in the detection of low-frequency somatic variants (at least 5–10% prevalence required) and requires optimization for high-quality sequences. NGS offers higher sensitivity to detect low-frequency variants [37,38], a faster turnaround time for high sample volumes [39], and a lower limit of detection [40,41]. Moreover, NGS enables the analysis of hundreds of thousands of reads per sample. The identification of somatic mutations, as shown here, improves our understanding of molecular pathogenesis in uPA. Further studies with NGS performed at CYP11B2-immunohistochemistry positive peri-tumoral areas (APN/APM) to confirm the clinical correlations to the mutation status are warranted.

Several limitations should also be acknowledged. First, most of our cNGS-identified mutations were detected in a small number of patients, and, therefore, comprehensive clinical and biological correlations of each of these rare mutations could not be analyzed. Second, a more targeted method of capturing *CYP11B2*-expressing tumor cells followed by sequencing was recently proposed, which increased the frequency of *CACNA1D* and *ATP1A1* somatic mutations [42]. We did not employ this targeted approach to guide DNA extraction and hence may have underestimated the prevalence of somatic mutations in uPA patients, especially those mutations in APN/APM. Third, the TAIPAI cohort evaluated here included patients with similar ethnic backgrounds. Our findings need to be confirmed in a broader population, including various ethnic groups. Fourth, novel mutations identified by cNGS in our study were not functionally characterized. As for some of the newly identified mutations, the locations of affected residues are different from previously reported mutation hotspots. For example, most of the previously reported somatic ATP2B3 mutations are located in the M4 transmembrane helix, whereas the newly identified variant (ATP2B3 p. Gly1189Ser) lies in the cytoplasmic domain. Further in vitro studies are required to investigate the potential roles and mechanisms of these newly identified mutations. Finally, genotype-clinical outcome associations need to be investigated using larger sample sizes. Prospective studies evaluating the predictive value of NGS mutation screening for post-adrenalectomy clinical outcomes in APA patients are also necessary.

## 5. Conclusions

Comprehensive customized and targeted NGS analysis in addition to Sanger sequencing identified somatic mutations in aldosterone-driver genes in 77.5% of uPA tumors, a higher incidence than that reported in previous studies on uPA using conventional approaches. Identification of mutation status may facilitate the prediction of better long-term clinical outcomes after adrenalectomy. Several novel mutations in aldosterone-driver genes were identified by cNGS in our cohort. The significance and function of these mutations require investigation in future studies.

## Figures and Tables

**Table 1:** Somatic mutations identified in unilateral primary aldosteronism by customized targeted next-generation sequencing.

No.	NGS-Identified Mutated Gene	Chr	Ref	Alt	Amino Acid Change	VAF	CADD Score	M-C	M-C Pred	PP2 Score	PP2_ Pred	MSC-PP2 Score	MSC-PP2 Pred	SIFT Score	SIFT Pred
**1**	ATP1A1	1	C	A	Leu670Ile	5.07	25.9	17.8	high	0.978	P	0.239	high	0.01	D
**2**	ATP2B3	X	G	A	Gly1189Ser	12.56	25.7	3.313	high	0.901	P	0.239	high	0	n/a
**3**	ATP2B3	X	G	A	Gly1189Ser	12.56	25.7	3.313	high	0.901	P	0.239	high	0	n/a
**4**	ATP2B3	X	T	C	Phe868Leu	5.15	24.7	3.313	high	0.415	benign	0.239	high	n/a	n/a
**5**	CACNA1D	3	G	A	Val1373Met	5.59	33	16.36	high	1	P	0.57	high	0	D
**6**	CACNA1D	3	G	A	Val1373Met	20.35	33	16.36	high	1	P	0.57	high	0	D
**7**	CACNA1D	3	C	T	Ser672Leu	39.75	35	16.36	high	0.992	P	0.57	high	0	D
**8**	CACNA1D	3	C	T	Arg240Cys	5.58	35	16.36	high	1	P	0.57	high	0	D
**9**	CACNA1D	3	G	A	Met590Ile	5.56	30	16.36	high	0.917	P	0.57	high	0	D
**10**	CACNA1D	3	T	C	Leu248Pro	5.14	28	16.36	high	1	P	0.57	high	0	D
**11**	CACNA1D	3	G	A	Ala2109Thr	8.25	28.7	16.36	high	0.999	P	0.57	high	0	D
**12**	CACNA1D	3	T	C	Val123Ala	5.45	26.4	16.36	high	0.999	P	0.57	high	0.02	D
**13**	CACNA1D	3	G	A	Gly1562Arg	5.60	34	16.36	high	0.986	P	0.57	high	0	D
**14**	CACNA1H	16	C	T	Pro277Ser	48.37	24.4	0.001	high	0.971	P	0.001	high	0.03	D
**15**	CACNA1H	16	A	G	Gln875Arg	8.03	25.9	0.001	high	0.830	P	0.001	high	0	D
**16**	CACNA1H	16	A	G	Thr615Pro	10.45	25.3	0.001	high	0.766	P	0.001	high	0	D
**17**	CACNA1H	16	C	T	Gln904Ter	10.20	38	0.001	high	n/a	n/a	0.001	n/a	0	D
**18**	CACNA1H	16	G	A	Trp482Ter	13.54	36	0.001	high	n/a	n/a	0.001	n/a	0.17	T
**19**	CLCN2	3	C	G	Gly466Ala	37.01	25.5	0.001	high	0.489	P	0.02	high	0	D
**20**	CLCN2	3	A	G	Phe252Ser	17.33	28.3	0.001	high	0.997	P	0.02	high	0	D
**21**	CTNNB1	3	C	T	Gln123Ter	7.87	37	16.39	high	n/a	n/a	0.239	n/a	0.01	D

**Table 2:** Clinical characteristics of unilateral primary aldosteronism patients with respect to NGS-identified mutations.

	All	cNGS-Identified Mutation Carrier	Cngs-Identified Mutation Non-Carrier	*p* Value (Mutation Carrier vs. Non-Carrier)
**Number**	75	21	54	
**Female (%)**	43 (57.3%)	14 (66.7%)	29 (53.7%)	0.436
**Age (years)**	54.5 (10.6)	53.5 (10.4)	54.9 (10.7)	0.603
**BMI (kg/m^2^)**	26.2 (4.0)	25.3 (3.4)	26.5 (4.2)	0.266
**sBP (mmHg)**	152.0 (20.8)	145.0 (19.9)	154.6 (20.7)	0.071
**dBP (mmHg)**	90.8 (14.5)	88.3 (17.4)	91.8 (13.3)	0.345
**PRA (ng/mL per min)** **†**	0.83 (2.32)	1.32 (4.07)	0.64 (1.06)	0.455
**Plasma aldosterone (ng/dL)** **†**	49.8 (35.2)	47.1 (29.9)	50.8 (37.2)	0.68
**Log ARR** **†**	2.28 (0.83)	2.33 (0.96)	2.26 (0.79)	0.742
**Potassium (mmol/L)** **†**	3.6 (0.6)	3.7 (0.6)	3.5 (0.6)	0.155
**Serum creatinine (mg/dL)**	0.93 (0.36)	0.85 (0.30)	0.95 (0.38)	0.279
**Cystatin C (mg/L)**	0.84 (0.23)	0.75 (0.25)	0.87 (0.30)	0.246
**Microalbuminuria (g/day)**	0.06 (0.12)	0.07 (0.13)	0.05 (0.11)	0.546
**Tumor size (cm)**	2.09 (2.57)	2.28 (2.98)	2.02 (2.43)	0.695
**baPWV (cm/s)**	1722.0 (329.2)	1647.5 (256.4)	1765.2 (361.8)	0.231
**Clinical success (%)**				0.010
**Complete**	33 (44%)	15 (71.4%)	18 (33.3%)	
**Absent**	16 (21.3%)	3 (14.3%)	13 (24.1%)	
**Partial**	26 (34.7%)	3 (14.3%)	23 (42.6%)	
**Biochemical success (%)**				0.440
**Complete**	61 (81.3%)	18 (85.7%)	43 (79.6%)	
**Absent**	10 (13.3%)	3 (14.3%)	7 (13%)	
**Partial**	4 (5.3%)	0	4 (7.4%)	

Abbreviations: ARR, aldosterone-renin ratio; BMI, body mass index; cNGS, customized targeted next-generation sequencing; dBP, diastolic blood pressure; PRA, plasma renin activity; PWV, pulse wave velocity; sBP, systolic blood pressure; Data are expressed as mean (standard deviation) unless otherwise indicated. † Obtained after hold drug that will interfere with the renin-angiotensin system. Definition of postoperative outcome: evaluation was performed at 12 months postoperatively. Clinical success was defined as resolution of hypertension and no need for anti-hypertensive medications. Complete biochemistry success was defined as aldosterone-to-plasma renin activity ratio <38 and serum potassium ≥3.5.

**Table 3:** Likelihood Ratio Tests of variables related to complete clinical success by multinomial logistic regression analysis.

	Model Fitting Criteria			Likelihood Ratio Tests	
	AIC of Reduced Model	BIC of Reduced Model	−2 Log Likelihood of Reduced Model	Chi-Square	Sig.
**Age** **(yr)**	165.545	216.530	121.545	1.825	0.402
**BMI** **(kg/m^2^)**	164.742	215.727	120.742	1.022	0.600
**SBP** **(mmHg)**	163.969	214.954	119.969	0.249	0.883
**DBP** **(mmHg)**	167.660	218.645	123.660	3.940	0.139
**Aldosterone** **(ng/dL) †**	167.252	218.237	123.252	3.532	0.171
**PRA** **(ng/mL/h) †**	165.395	216.380	121.395	1.675	0.433
**Potassium** **(mEq/L) †**	164.553	215.538	120.553	0.833	0.659
**Cr (mg/dL)**	165.379	216.364	121.379	1.659	0.436
**Tumor size (cm)**	168.261	219.246	124.261	4.541	0.103
**cNGS-identified mutation (yes)**	172.646	223.631	128.646	8.926	0.012
**Sex** **(Female)**	168.601	219.586	124.601	4.881	0.087

Abbreviations: BMI, body mass index; Cr, Serum creatinine; dBP, diastolic blood pressure; PRA, plasma renin activity; SBP, systolic blood pressure; The chi-square statistic is the difference in -2 log-likelihoods between the final model and a reduced model. The reduced model is formed by omitting an effect from the final model. The null hypothesis states all parameters of that effect are 0. Tumor size was measured using computed tomography or magnetic resonance imaging. † Obtained after hold drug that will interfere the renin-angiotensin system.

## Data Availability

The data presented in this study are available on request from the authors.

## Supplementary Materials

The following are available online at https://www.mdpi.com/article/10.3390/biomedicines9091167/s1, Table S1. Primers used for Sanger sequencing, Table S2. The definition of clinical and biochemical outcome according to the PASO consensus, Table S3. Clinical characteristics of unilateral primary aldosteronism patients with respect to [NGS + Sanger]-identified mutations, Table S4. Likelihood Ratio Tests of variables related to complete clinical success by multinomial logistic regression analysis, Table S5. Likelihood Ratio Tests of cNGS-identified mutation related to complete clinical success by multinomial logistic regression analysis with models adjusted for different clinical co-variables, Table S6. Likelihood Ratio Tests of Sanger sequencing-identified mutation related to complete clinical success by multinomial logistic regression analysis, Table S7. Likelihood Ratio Tests of cNGS-identified mutation related to complete clinical success by multinomial logistic regression analysis among 51 uPA with CYP11B2-expressing adrenocortical tumors, Figure S1. The diagnosis and treatment protocol of TAIPAI, Figure S2. Clinical outcome for 240 patients with unilateral primary aldosteronism who underwent adrenalectomy with respect to detected mutations via customized, targeted next-generation sequencing + Sanger sequencing, Figure S3. Characteristics of tumoral tissues of unilateral primary aldosteronism based on the results of CYP11B2 immunohistochemistry.
