# Supplementary material for: Novel Mutations Detection with Next-Generation Sequencing and Its Association with Clinical Outcome in Unilateral Primary Aldosteronism
Source: Biomedicines. 2021 Sep 6;9(9):1167. doi: 10.3390/biomedicines9091167 (PMC8471673; doi:10.3390/biomedicines9091167)
Supplement: Supplementary file 1 [file biomedicines-09-01167-s001.zip › biomedicines-1321403-supplementary.pdf]

Supplementary Material and methods

Patients were enrolled from the following hospitals:

Two medical centers (National Taiwan University Hospital (NTUH), Taipei, Taiwan; Taipei Medical University Hospital, Taipei, Taiwan) and five regional hospitals (Cardinal Tien Hospital, New Taipei City, Taiwan; Taipei Tzu Chi Hospital, New Taipei City, Taiwan; Yun- Lin Branch of NTUH, Douliou City, Taiwan; Hsin-Chu Branch of NTUH, Hsin-Chu City, Taiwan; Zhongxing Branch of Taipei City Hospital, Taipei, Taiwan)[1].

Our standard protocol to identify primary aldosteronism (PA) and functional laterization:

Hypertensive patients were diagnosed with primary aldosteronism based on the following criteria [2-4] (Fig S1):

Confirmation

Fulfillment of the following three conditions confirmed a diagnosis of PA: (1) autonomous excess aldosterone production evidenced by an aldosterone-renin ratio (ARR) > 35; (2) a TAIPAI score larger than 60% [5]; (3) seated post-saline loading PAC > 16 ng/dL, or PAC/PRA > 35 (ng/dL)/(ng/mL/h) shown in a post-captopril test [6]. (Abbreviations: PAC, plasma aldosterone concentration; PRA, plasma renin activity).

The probability of PA (TAIPAI score) was equal to:

$1 + e^{-\beta}$; where β = (PAC [ng/dl] × [0.063]) + PRA [ng/ml/h] × [−0.205]) + ([ARR × 0.001] + BMI [kg/m^2] × [0.067]) + (Male × [−0.738] + SK [mmol/l] × [−1.512]) + (eGFR [ml/min/1.73 m^2] × [0.017]) + ([propensity score] × [−0.539] + [1.851])

Lateralization

1. Unilateral primary aldosteronism (uPA) was identified on the basis on the following four criteria[7]:
   (a) Confirmed PA diagnosis;
   (b) Imaging evidence for a unilateral adrenal adenoma or hyperplasia;
   (c) Lateralization of aldosterone secretion with adrenal vein sampling (AVS) or during dexamethasone suppression NP-59 SPECT/CT[8] to the above-mentioned imagine-finding side.

2. Selectivity and lateralization indices of AVS
   The selectivity index (SI) is defined as the ratio of the sampled cortisol concentration of each adrenal vein to that of the peripheral vein. The lateralization index (LI) is defined as the ratio of the aldosterone/cortisol concentration on the dominant side to that on the contralateral side. Successful AVS is defined as an SI value ≥2.0 bilaterally. After confirming successful bilateral AVS, lateralization of the PA was determined by an LI value ≥2.0.[11]

Selectivity and lateralization indices of NP-59 SPECT/CT, here we summarize it as:

When the patients and/or the physician did not prefer or could not perform an AVS for PA lateralization, lateralization of aldosterone secretion was confirmed with a dexamethasone suppression 131I-6b-Iodomethyl-19-Norcholesterol (NP-59) SPECT/CT [8, 12] with the combination use of a 5-point visual scale (3 or 4 of 0~4 scale); , and semi-quantification of adrenal to liver ratio (ALR; cut-off value= 2.28) and lesion to contralateral ratio of bilateral adrenal glands (CON; cut-off value= 1.11) to lateralize the functional lesion to the side of the adrenal glands with the adenoma.

The total examination period for NP-59 scintigraphy is 8 days. To avoid the interruption of NP-59 uptake in adrenal glands, some anti-hypertensive medications should be held prior to NP-59 injection. Oral dexamethasone (8 mg daily, divided into two doses throughout the study) with a total dose of 64 mg is given to ensure maximal suppression of normal adrenal cortical uptake. One mL of diluted Lugol’s solution daily is also given throughout the study to protect the thyroid from free 131I uptake; 37 MBq (1 mCi) NP-59 is injected slowly via venous route on the fourth day of the study.

Planar imaging with anterior and posterior views is routinely performed at 72 h after NP-59 injection. Abdominal SPECT/CT is performed at 96 h after NP-59 injection if no obvious lesion is recognized by 72 h imaging.

Mild laxatives can be used each night before NP-59 imaging to reduce intestinal background activity. Image interpretation is usually done by a five-point visual scale (VS) of SPECT/CT. A scale value of 0 points represents no significant uptake by the adrenal gland, 1 point represents faint uptake less than or equal to liver uptake, 2 points represents bilaterally symmetric uptake slightly greater than liver uptake, 3 points represents focal unilateral or

asymmetric uptake greater than liver uptake and 4 points represents intense unilateral or asymmetric uptake significantly greater than liver uptake. An adrenal lesion is considered an APA if VS is either 3 or 4.2 Semiquantification by drawing regions of interest (ROI) of adrenal glands and liver on the transaxial view of SPECT/CT provides better ability to differentiate APA from IAH. Semiquantification also provides a predictive value of clinical outcome after adrenalectomy[7].

Material and methods

The concentration of aldosterone was measured with a radioimmunoassay using commercial kits (Aldosterone Maia Kit; Adaltis Italia S.r.l., Bologna, Italy). The lowest detectable concentration of aldosterone was 1.0 ng/dL. The normal range of aldosterone is 7–35 ng/dL with the patient in the upright position. Plasma renin activity (PRA) was measured via the generation of angiotensin I in vitro, using a commercially available radioimmunoassay kit (Stillwater, MN, USA). The normal range of PRA is 2.63 ± 1.32 ng/mL/ h with the patient in the upright position. The mean (standard deviation [SD]) intra- and interassay coefficients of variation for the PRA assay were 1.9 (5.0%) and 4.5 (5.2%), respectively.

Targeted Next-Generation Sequencing

1. NGS was made in-house. Primers were ordered from Integrated DNA Technologies IDT, Inc.
2. Tools used to design the panel:
   i. UCSC Genome Browser on Human Feb. 2009 (GRCh37/hg19) Assembly: https://genome.ucsc.edu/cgi-bin/hgTracks?db=hg19&lastVirtModeType=default&lastVirtModeExtraState=&virtModeType=default&virtMode=0&nonVirtPosition=&position=chrX%3A15578261%2D15621068&hgsid=1144608959_9JwbUaZAIABu5TyYWIrW03hc6Hzh
   ii. Primer-BLAST: https://www.ncbi.nlm.nih.gov/tools/primer-blast/
3. Primers per gene:
   KCNJ5: 7; ATP1A1: 27; ATP2B3: 31; CACNA1D: 55; CACNA1H: 47; CLCN2: 24; CTNNB1: 23 (Total primer pairs: 214)
4. Total size of the target region: 55,909bp
5. MiniSeq was used with High Output Reagent 2x150 kit. Read length was

set as: 151+10+151. 96 samples were pooled in one run without PhiX (balanced library composed of more than 200 amplicon).

6. First run: 96 sample; cluster density: 337±2; PF: 75.99±0.61; Q30: 86.66;
7. Second run: 96 sample; cluster density: 162±2; PF: 94.72±0.24; Q30: 95.24

Table S1. Primers used for Sanger sequencing

KCNJ5 **(NM_000890.5)**			**Detectable mutations**	**Base pair**
KCNJ5 GC[13]	Forward	CTTCATTTGGTGGCTCATTGC	p.Thr148Ile<br>p.Thr149Ser<br>p.Thr148_Thr149insArg<br>p.Thr149dup<br>p.Gly151Glu<br>p.Gly151Arg<br>p.Tyr152Cys<br>p.Phe154Gys<br>p.Ile157del<br>p.Ile157Ser<br>p.Thr158Ala<br>p.Leu168Arg	410
	Reverse	GGACTTGATGAGCTTGGC		
KCNJ5 G2[13]	Forward	CTTTCCTGTTCTCCATTGAGACC	p.Glu246Gly	544
	Reverse	CTGAGGAGGACAAAGCGCC		

ATP2B3 **(NM_001001344.2)**			**Detectable mutations**	**Base pair**
ATP2B3-Exon 8	Forward	CCTGGGCTGTTTATCCTGAA	p.Val424_Leu425del<br>p.Leu425_Val426del<br>p.Val426_Val427del	416
	Reverse	CCCCAGTTTCCGAGTCTGTA		

CTNNB1 **(NM_001904.4)**			**Detectable mutations**	**Base pair**
CTNNB1-Exon[14]	Forward	CATTCTGCTTTTCTTGGCTGTC	p.Ser33Cys<br>p.Gly34Arg<br>p.Ser45Phe<br>p.Ser45Pro<br>p.Ser45Cys	483
	Reverse	GCTATTACTCTCTTTTCTTCCC		

CLCN2 **(NM_004366.6)**			**Detectable mutations**	**Base pair**
CLCN2 -Exon 2[15]	Forward<br>Reverse	GTGGG AACTG TGCGT TCAAG<br>AGCAG CTCTA ATGGC CTCTG	p.Gly24Asp<br>p.Met22Lys<br>p.Tyr26Asn	360
CLCN2-Exon 5[16]	Forward<br>Reverse	GGACC CCTGA AATGG GTGCA TCG<br>TGAGT CGGGA GGGGG CCCGC CCTG	p.Arg172Gln	273

ATP1A1 **(NM_001160233.2)**			**Detectable mutations**	**Base pair**
ATP1A1-Exon 4	Forward<br>Reverse	TTCCTTGGGCCTATTGTTTG<br>GTGGGAGACA AAGACGGAGA	p.Gly99 Arg<br>p.Leu104Arg<br>p.Phe100_Leu104del<br>p.Met102_Leu103del<br>p.Met102_Ile106del<br>p.leu103_Leu104del	487
ATP1A1-Exon 8	Forward<br>Reverse	CGTGGCTTCCTTCAGGTTAG<br>CGTGATGTGGCTCTCAAGAA	p.Val332Gly	468

Table S2. The definition of clinical and biochemical outcome according to the PASO consensus [17]

Outcome measure	Clinical outcome definition	Biochemical outcome definition
**Complete success (remission)**	Normal BP without the aid of antihypertensive medication	Correction of hypokalemia and normalization of the aldosterone-to-renin ratio
**Partial success (improvement)**	The same BP with less antihypertensive medication ***or***<br>A reduction in BP with either the same amount or less antihypertensive medication	Correction of hypokalemia and a raised aldosterone-to-renin ratio with one or both of the following (compared with pre-surgery): ≥50% decrease in baseline plasma aldosterone concentration; or abnormal but improved post-surgery confirmatory test result.
**No (absence of) success (persistence)**	Unchanged or increased BP levels with either the same amount or an increase in antihypertensive medication	Persistent hypokalemia or persistent raised aldosterone-to-renin ratio, or both, with failure to suppress aldosterone secretion with a post-surgery confirmatory test.

Table S3. Clinical characteristics of unilateral primary aldosteronism patients with respect to [NGS + Sanger]-identified mutations

	All	Mutation carrier	Mutation non-carrier	p value (mutation carrier vs. non-carrier)
**Number**	240	186	54	
**Female(%)**	134(55.8%)	105(56.5%)	29(53.7%)	0.876
**Age (years)**	49.7(10.9)	48.5(11.0)	54.9(10.7)	0.001
**BMI (kg/m^2)**	25.4(4.2)	25.2(4.2)	26.5(4.2)	0.08
**sBP (mmHg)**	153.5(22.8)	153.2(23.4)	154.6(20.7)	0.689
**dBP (mmHg)**	91.9(15.4)	91.9(16.0)	91.8(13.3)	0.833
**PRA (ng/ml per min) †**		0.79(3.38)	0.64(1.06)	0.741
**Plasma aldosterone (ng/dl) †**	62.4(47.6)	65.8(49.8)	50.8(37.2)	0.045
**Log ARR†**	2.41(0.80)	2.44(0.80)	2.26(0.79)	0.241
**Potassium (mmol/L) †**	3.4(0.7)	3.3(0.7)	3.5(0.6)	<0.0001
**Serum creatinine (mg/dl)**	0.91(0.37)	0.90(0.37)	0.95(0.38)	0.480
**Tumor size (cm)**	1.83(1.23)	1.76(0.63)	2.02(2.43)	0.363
**Clinical success¶ (%)**				<0.0001
**Complete**	148(61.7%)	130(69.9%)	18(33.3%)	
**Absent**	26(10.8%)	13(7.0%)	13(24.1%)	
**Partial**	66(27.5%)	43(23.1%)	23(42.6%)	
**Biochemical success¶ (%)**				0.460
**Complete**	206(85.8%)	163(87.6%)	43(79.6%)	
**Absent**	22(9.2%)	15(8.1%)	7(13%)	
**Partial**	12(5%)	8(4.3%)	4(7.4%)	

Abbreviations: ARR, aldosterone-renin ratio; BMI, body mass index; cNGS, customized targeted next-generation sequencing; dBP, diastolic blood pressure; PRA, plasma renin activity; PWV, pulse wave velocity; sBP, systolic

blood pressure; *Data are expressed as mean (standard deviation) unless otherwise indicated.

†Obtained after hold drug that will interfere the renin-angiotensin system.

¶ Definition of postoperative outcome: Evaluation was performed at 12 months postoperatively. Clinical success was defined as resolution of hypertension and no need for antihypertensive medications. **Complete biochemistry success** was defined as aldosterone-to-plasma renin activity ratio <38 and serum potassium ≥3.5.

Table S4. Likelihood Ratio Tests of variables related to complete clinical success by multinomial logistic regression analysis§

	Model Fitting Criteria			Likelihood Ratio Tests	
	AIC of Reduced Model	BIC of Reduced Model	-2 Log Likelihood of Reduced Model	Chi-Square	Sig.
**Age(yr)**	397.961	473.599	353.961	10.872	.004
**BMI (kg/m^2)**	392.149	467.787	348.149	5.060	.080
**SBP(mmHg)**	390.104	465.742	346.104	3.015	.221
**DBP(mmHg)**	390.549	466.186	346.549	3.460	.177
**Aldosterone(ng/dL) †**	388.478	464.116	344.478	1.389	.499
**PRA(ng/mL/hr) †**	389.666	465.304	345.666	2.577	.276
**Potassium(mEq/L) †**	395.096	470.734	351.096	8.007	.018
**Cr (mg/dL)**	388.598	464.236	344.598	1.509	.470
**Tumor size (cm)¶**	389.196	464.833	345.196	2.107	.349
**[NGS+Sanger]-*identified* mutation (yes)**	398.312	473.950	354.312	11.223	.004
**Sex (Female)**	387.789	463.427	343.789	.700	.705

Abbreviations: BMI, body mass index; Cr, Serum creatinine; dBP, diastolic blood pressure; PRA, plasma renin activity; sBP, systolic blood pressure;

§The chi-square statistic is the difference in -2 log-likelihoods between the final model and a reduced model. The reduced model is formed by omitting an effect from the final model. The null hypothesis states all parameters of that effect are 0.

¶ Tumor size was measured using computed tomography or magnetic resonance imaging.

†Obtained after hold drug that will interfere the renin-angiotensin system.

Table S5. Likelihood Ratio Tests of cNGS-identified mutation related to complete clinical success by multinomial logistic regression analysis with models adjusted for different clinical co-variables

		Model I	Model II	Model III	Model IV	Model V
**Model Fitting Criteria**	AIC of Reduced Model	163.106	168.688	171.898	170.488	172.646
	BIC of Reduced Model	186.281	201.133	208.978	216.837	223.631
	-2 Log Likelihood of Reduced Model	143.106	140.688	139.898	130.488	128.646
**Likelihood Ratio Tests of cNGS-identified mutation (yes)**	Chi-Square	8.931	10.257	10.582	9.108	8.926
	Sig.	0.012	0.006	0.005	0.011	0.012

Model I: adjusted by sex, age, BMI, and tumor size
Model II: adjusted by sex, age, BMI, tumor size, PAC, and PRA
Model III: adjusted by sex, age, BMI, tumor size, PAC, PRA, and serum potassium
Model IV: adjusted by sex, age, BMI, tumor size, SBP, DBP, PAC, PRA, and serum potassium
Model V: adjusted by sex, age, BMI, tumor size, SBP, DBP, PAC, PRA, serum potassium, and serum Cr

Table S6. Likelihood Ratio Tests of Sanger sequencing-identified mutation related to complete clinical success by multinomial logistic regression analysis§

	Model Fitting Criteria			Likelihood Ratio Tests	
	AIC of Reduced Model	BIC of Reduced Model	-2 Log Likelihood of Reduced Model	Chi-Square	Sig.
**Age(yr)**	369.404	443.455	325.404	13.955	.001
**BMI (kg/m^2)**	361.911	435.963	317.911	6.463	.039
**SBP(mmHg)**	358.197	432.248	314.197	2.749	.253
**DBP(mmHg)**	360.159	434.210	316.159	4.711	.095
**Aldosterone(ng/dL) †**	356.149	430.201	312.149	0.701	.704
**PRA(ng/mL/hr) †**	356.491	430.543	312.491	1.043	.594
**Potassium(mEq/L) †**	362.437	436.489	318.437	6.989	.030
**Cr (mg/dL)**	358.646	432.697	314.646	3.197	.202
**Tumor size (cm)¶**	357.499	431.551	313.499	2.051	.359
**Sanger sequencing *-identified* mutation (vs. mutation negative)**	363.867	437.919	319.867	8.419	.015
**Sex (Female)**	356.393	430.444	312.393	0.944	.624

Abbreviations: BMI, body mass index; Cr, Serum creatinine; dBP, diastolic blood pressure; PRA, plasma renin activity; sBP, systolic blood pressure;

§The chi-square statistic is the difference in -2 log-likelihoods between the final model and a reduced model. The reduced model is formed by omitting an

effect from the final model. The null hypothesis states all parameters of that effect are 0.

¶ Tumor size was measured using computed tomography or magnetic resonance imaging.

†Obtained after hold drug that will interfere the renin-angiotensin system.

Table S7. Likelihood Ratio Tests of cNGS-identified mutation related to complete clinical success by multinomial logistic regression analysis among 51 uPA with CYP11B2-expressing adrenocortical tumors§

	Model Fitting Criteria			Likelihood Ratio Tests	
	AIC of Reduced Model	BIC of Reduced Model	-2 Log Likelihood of Reduced Model	Chi-Square	Sig.
**Age(yr)**	84.747	119.584	40.747	2.284	.319
**BMI (kg/m^2)**	88.083	122.920	44.083	5.620	.060
**SBP(mmHg)**	82.784	117.621	38.784	.321	.852
**DBP(mmHg)**	83.987	118.824	39.987	1.524	.467
**Aldosterone(ng/dL) †**	87.131	121.968	43.131	4.668	.097
**PRA(ng/mL/hr) †**	86.521	121.359	42.521	4.058	.131
**Potassium(mEq/L) †**	84.511	119.348	40.511	2.048	.359
**Cr (mg/dL)**	84.878	119.716	40.878	2.415	.299
**Tumor size (cm)¶**	82.590	117.427	38.590	.127	.939
**cNGS-*identified* mutation (yes)**	94.494	129.331	50.494	12.031	.002
**Sex (Female)**	87.092	121.929	43.092	4.629	.099

Abbreviations: BMI, body mass index; Cr, Serum creatinine; dBP, diastolic blood pressure; PRA, plasma renin activity; sBP, systolic blood pressure;

§The chi-square statistic is the difference in -2 log-likelihoods between the final model and a reduced model. The reduced model is formed by omitting an effect from the final model. The null hypothesis states all parameters of that effect are 0.

¶ Tumor size was measured using computed tomography or magnetic resonance imaging.
†Obtained after hold drug that will interfere the renin-angiotensin system.

Figure S1. The diagnosis and treatment protocol of TAIPAI. [3, 6, 10].

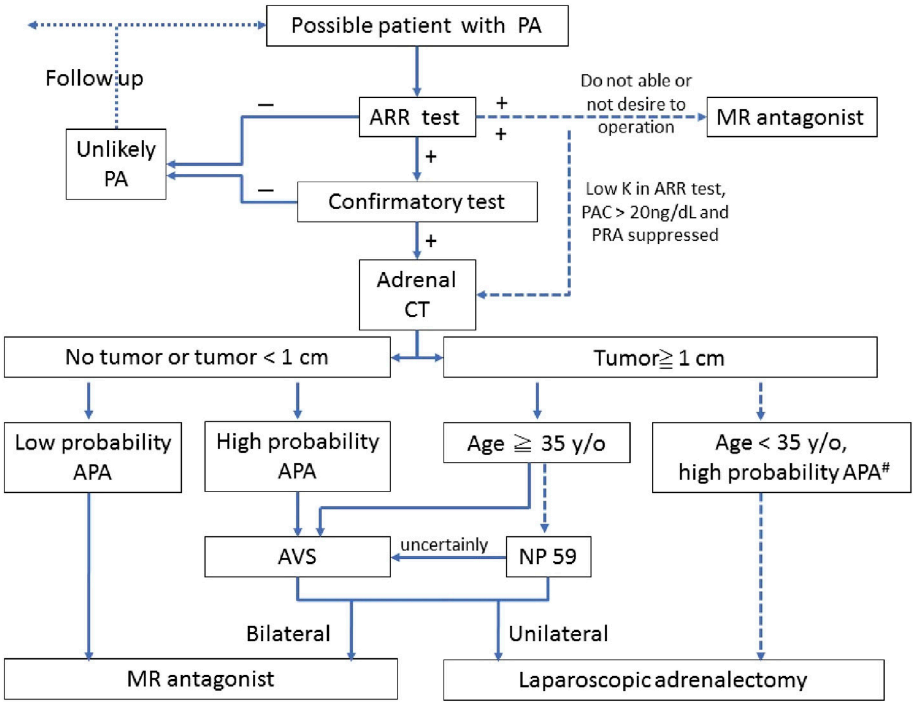

Patients < 35 years with adrenal lesion S 1 cm can also receive AVS if clinically indicated.

Abbreviations: APA, aldosterone-producing adenomas; ARR, aldosterone-to-renin ratio; AVS, adrenal venous sampling; CT, computed tomography; NP-59- SPECT, I-131-6-beta-iodomethyl-19-norcholesterol single-photon emission

computed tomography

Figure S2. Clinical outcome for 240 patients with unilateral primary aldosteronism who underwent adrenalectomy with respect to detected mutations via customized targeted next-generation sequencing + Sanger sequencing.

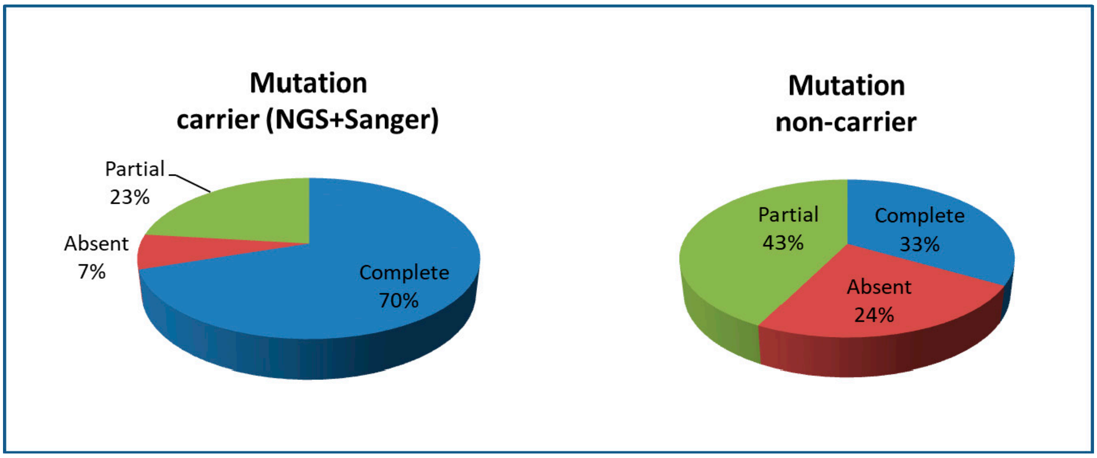

¶ Definition of postoperative outcome: Evaluation was performed at 12 months postoperatively. Clinical success was defined as resolution of hypertension and no need for antihypertensive medications.

Figure S3. Characteristics of tumoral tissues of unilateral primary aldosteronism based on the results of CYP11B2 immunohistochemistry

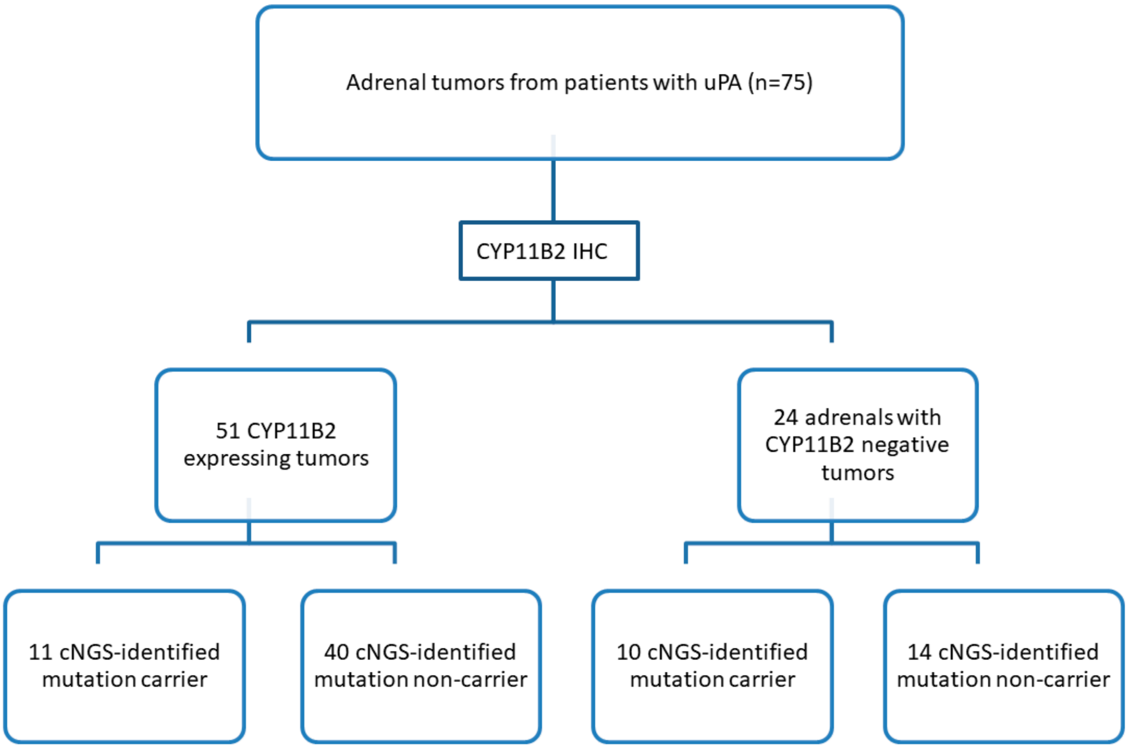

References

1. Wu VC, Hu YH, Wu CH, Kao CC, Wang CY, Yang WS, Lee HH, Chang YS, Lin YH, Wang SM *et al*: **Administrative data on diagnosis and mineralocorticoid receptor antagonist prescription identified patients with primary aldosteronism in Taiwan**. *J Clin Epidemiol* 2014, **67**(10):1139-1149.
2. Wu VC, Chang HW, Liu KL, Lin YH, Chueh SC, Lin WC, Ho YL, Huang JW, Chiang CK, Yang SY *et al*: **Primary Aldosteronism: Diagnostic Accuracy of the Losartan and Captopril Tests**. *Am J Hypertens* 2009, **22**(8):821-827.
3. Kuo CC, Wu VC, Huang KH, Wang SM, Chang CC, Lu CC, Yang WS, Tsai CW, Lai CF, Lee TY *et al*: **Verification and evaluation of aldosteronism demographics in the Taiwan Primary Aldosteronism Investigation Group (TAIPAI Group)**. *J Renin Angiotensin Aldosterone Syst* 2011, **12**(3):348-357.
4. Wu VC, Kuo CC, Wang SM, Liu KL, Huang KH, Lin YH, Chu TS, Chang HW, Lin CY, Tsai CT *et al*: **Primary aldosteronism: changes in cystatin C-based kidney filtration, proteinuria, and renal duplex indices with treatment**. *J Hypertens* 2011, **29**(9):1778-1786.
5. Wu VC, Yang SY, Lin JW, Cheng BW, Kuo CC, Tsai CT, Chu TS, Huang KH, Wang SM, Lin YH *et al*: **Kidney impairment in primary aldosteronism**. *Clin Chim Acta* 2011, **412**(15-16):1319-1325.
6. Chao CT, Wu VC, Kuo CC, Lin YH, Chang CC, Chueh SJ, Wu KD, Pimenta E, Stowasser M: **Diagnosis and management of primary aldosteronism: an updated review**. *Annals of medicine* 2013, **45**(4):375-383.
7. Wu VC, Hu YH, Er LK, Yen RF, Chang CH, Chang YL, Lu CC, Chang CC, Lin JH, Lin YH *et al*: **Case detection and diagnosis of primary aldosteronism - The consensus of Taiwan Society of Aldosteronism**. *J Formos Med Assoc* 2017, **116**(12):993-1005.
8. Yen RF, Wu VC, Liu KL, Cheng MF, Wu YW, Chueh SC, Lin WC, Wu KD, Tzen KY, Lu CC: **131I-6beta-iodomethyl-19-norcholesterol SPECT/CT for primary aldosteronism patients with inconclusive adrenal venous sampling and CT results**. *J Nucl Med* 2009, **50**(10):1631-1637.
9. Wu VC, Wang SM, Chueh SJ, Yang SY, Huang KH, Lin YH, Wang JJ, Connolly R, Hu YH, Gomez-Sanchez CE *et al*: **The prevalence of CTNNB1 mutations in primary aldosteronism and consequences for clinical outcomes**. *Sci Rep* 2017, **7**:39121.
10. Wu VC, Chao CT, Kuo CC, Lin YH, Chueh SC, Wu KD: **Diagnosis and Management of Primary Aldosteronism**. *Acta Nephrologica* 2012, **26**(3):111-120.

11. Chang CC, Lee BC, Chang YC, Wu VC, Huang KH, Liu KL, Group TS: **Comparison of C-arm computed tomography and on-site quick cortisol assay for adrenal venous sampling: A retrospective study of 178 patients**. *Eur Radiol* 2017, **27**(12):5006-5014.
12. Lu CC, Wu VC, Wu KD, Liu KL, Lin WC, Cheng MF, Tzen KY, Yen RF, Group TS: **Prognostic value of semiquantification NP-59 SPECT/CT in primary aldosteronism patients after adrenalectomy**. *Eur J Nucl Med Mol Imaging* 2014, **41**(7):1375-1384.
13. Azizan EA, Murthy M, Stowasser M, Gordon R, Kowalski B, Xu S, Brown MJ, O'Shaughnessy KM: **Somatic mutations affecting the selectivity filter of KCNJ5 are frequent in 2 large unselected collections of adrenal aldosteronomas**. *Hypertension* 2012, **59**(3):587-591.
14. Teo AE, Garg S, Shaikh LH, Zhou J, Karet Frankl FE, Gurnell M, Happerfield L, Marker A, Bienz M, Azizan EA *et al*: **Pregnancy, Primary Aldosteronism, and Adrenal CTNNB1 Mutations**. *N Engl J Med* 2015, **373**(15):1429-1436.
15. Fernandes-Rosa FL, Daniil G, Orozco IJ, Goppner C, El Zein R, Jain V, Boulkroun S, Jeunemaitre X, Amar L, Lefebvre H *et al*: **A gain-of-function mutation in the CLCN2 chloride channel gene causes primary aldosteronism**. *Nat Genet* 2018, **50**(3):355-361.
16. Scholl UI, Stolting G, Schewe J, Thiel A, Tan H, Nelson-Williams C, Vichot AA, Jin SC, Loring E, Untiet V *et al*: **CLCN2 chloride channel mutations in familial hyperaldosteronism type II**. *Nat Genet* 2018, **50**(3):349-354.
17. Williams TA, Lenders JWM, Mulatero P, Burrello J, Rottenkolber M, Adolf C, Satoh F, Amar L, Quinkler M, Deinum J *et al*: **Outcomes after adrenalectomy for unilateral primary aldosteronism: an international consensus on outcome measures and analysis of remission rates in an international cohort**. *Lancet Diabetes Endocrinol* 2017, **5**(9):689-699.
